# Patient-Centric Paradigm: A Systems Thinking Approach to Enhance Healthcare

**DOI:** 10.3390/healthcare13030213

**Published:** 2025-01-21

**Authors:** Ashiat Adeogun, Misa Faezipour

**Affiliations:** 1 Healthcare Informatics Program, College of Basic and Applied Sciences, Middle Tennessee State University, Murfreesboro, TN 37132, USA; aaa2ev@mtmail.mtsu.edu; 2 Department of Engineering Technology, Middle Tennessee State University, Murfreesboro, TN 37132, USA

**Keywords:** patient-centric approaches, causal model loop, systems thinking, system dynamics, healthcare system, social determinants of health

## Abstract

**Objective:** The study aims to investigate the impact of patient-centric approaches on patient health outcomes, identify key leverage points for enhancing patient-centered care, and evaluate the effectiveness of systems thinking in guiding healthcare transformations. **Methods and Procedures:** The research utilizes system dynamics methodology, combining qualitative and quantitative data with causal loop diagrams and simulation models. Using Vensim software, the study examines dynamic interactions, feedback loops, and the effects of patient-centric interventions. Sensitivity analysis assesses the impact of variables such as access to healthcare improvements, and social determinants of health (SDOH), providing insights into the systemic behaviors of healthcare models. **Results:** Simulation analyses demonstrate the effectiveness of patient-centric interventions in improving engagement, satisfaction, communication, and health outcomes. Key leverage points, such as enhanced patient–provider communication and addressing SDOH, are critical for driving sustainable improvements. However, declining trends in outcomes over time indicate the need for adaptive strategies to maintain effectiveness. **Conclusions:** The study emphasizes the importance of maintaining patient centricity in healthcare settings. By leveraging systems thinking and addressing underlying factors such as SDOH, the research provides actionable insights to enhance care delivery and patient outcomes. Despite the limitations of simulated data, the findings contribute to understanding the dynamic interplay between patient-centric strategies and healthcare system performance, advocating for sustained efforts to ensure equitable and effective care.

## 1. Introduction

Patient centricity is a simple idea that encompasses integrated actions to actively listen to and interact with patients, putting their well-being first in all initiatives [1]. Several factors contribute to the prevalence of patient centricity nowadays. This could be a reaction to a changing landscape in which patients are more connected and well informed. This could also represent an acknowledgment of the ethical responsibilities inherent in healthcare settings [2,3].

The literature extensively examines initiatives to improve the efficiency and value of healthcare services. Patients are increasingly recognized as essential stakeholders in the healthcare dialogue: regulatory agencies such as the Food and Drug Administration (FDA) and the European Medicines Agency (EMA), academia, and healthcare providers are asking for patient input in their decision-making [4].

Additionally, this approach represents a holistic strategy for disease management. Despite widespread recognition of the value of patient-centeredness, there is a notable lack of published guidance, and its implementation within the pharmaceutical industry remains inconsistent [5]. With patients becoming more informed and engaged, the demand for personalized care is rising, emphasizing the ethical responsibility of healthcare systems to prioritize patient needs, improve health outcomes, and foster trust. For businesses, this necessitates balancing commercial objectives with a genuine commitment to patient-centered decision-making and services [1].

In line with previous healthcare research, this article advocates for the use of systems thinking (ST), particularly through causal loop diagrams (CLDs) as demonstrated by [6,7] to address the dynamic complexities inherent in healthcare settings. The goal is to drive innovative changes that lead to more efficient, safe, and high-quality care. According to [8], the application of ST fosters conditions that allow local health professionals to innovate within their hospital units, thus improving community health and ultimately the broader health system.

The purpose of ST modeling extends beyond operational improvements; it seeks to reshape how we conceptualize systems, offering significant potential to influence policy [9], promote learning and challenge existing mental models [10]. Such models enable explicit learning opportunities, allowing individuals to test and refine their understanding of complex systems and processes, ultimately enhancing their ability to address challenges effectively.

**Figure 1:**
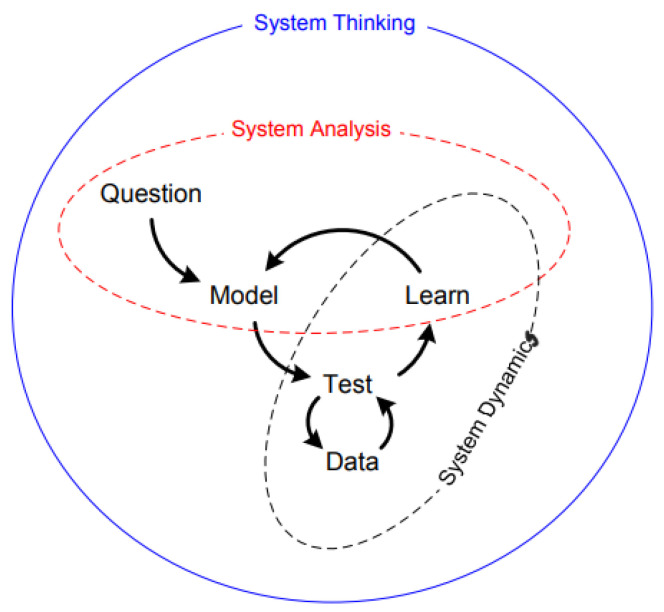
Understanding complexity through systems thinking and dynamics.

ST has evolved over the last 60 years and has increasingly impacted research. In a nutshell, ST is a science that works by organizing logic and the integration of disciplines to comprehend patterns and relationships in complicated issues. Science discovers links and relationships between seemingly disparate elements. ST incorporates two other concepts: systems analysis (SA) and system dynamics (SD). In general, ST is the mental modeling and science of structuring logic and asking pertinent questions, but this also has practical applications [11] as shown in Figure 1. SA is “the art and science of making reliable inferences about behavior by developing an increasingly deep understanding of the underlying structure [12]”.

SA focuses on identifying organizational structures within systems and gaining insights into the arrangement of causal relationships. This approach involves breaking down and reconstructing problems to understand their components and feedback mechanisms. It also incorporates group modeling, where the initial problem is explored, and a mental model structure is developed to represent the issue using CLDs [11].

SD is a computer-aided methodology designed for policy analysis and the resolution of complex problems. Rooted in control engineering and management, this approach adopts an information feedback and delay perspective to explore the dynamic patterns inherent in intricate biological, physical, and social systems [13].

A simulation model provides a powerful tool for studying system behavior as it evolves over time. Once constructed and validated, such a model can be applied to explore a wide range of “what-if” scenarios relevant to real-world systems.

The increasing complexities of contemporary biomedicine underscore the importance of modeling and simulation in understanding and predicting the progression of pathophysiology, the origins of diseases, and their spread. These tools are invaluable for both clinical and policy decision-making, enabling informed strategies and interventions [14]. By their nature, healthcare systems are highly intricate, composed of numerous interconnected and interdependent components with feedback mechanisms involving multiple independent agents interacting within a broader environment. Addressing this complexity necessitates the use of ST, which offers an effective framework for analyzing and understanding the multifaceted nature of health systems [15].

The healthcare system faces challenges such as fragmented care, a lack of patient engagement, and suboptimal communication among healthcare providers to the patients. These issues contribute to reduced patient satisfaction and suboptimal patient health outcomes. The study addresses these challenges by exploring how a systems thinking approach can reshape the healthcare landscape and address the root causes of these problems.

Moreover, ST has been recognized as a valuable tool in driving change within health service delivery, particularly in science implementation. It complements and improves traditional reductionist approaches to health improvement by addressing their limitations [8]. ST helps to resolve the recurring unintended consequences often associated with health policies. In addition, it facilitates a deeper understanding of health problems and contexts, enable more effective collaboration with stakeholders. By examining the overall functioning of healthcare systems [9], ST provides critical insights that contribute to enhancing the quality of healthcare delivery.

The following are the objectives of patient centricity:

1. Investigate the impact of patient-centric approaches on patient healthcare outcomes.
2. Identify the key leverage points within the healthcare system for enhancing patient-centric care.
3. Evaluate the effectiveness of ST in guiding healthcare transformations.

We hypothesize that implementing ST approach in healthcare will improve patient satisfaction, improve treatment adherence, and elevate overall patient health outcomes. By addressing the interconnections among diverse healthcare components, we aim to develop innovative strategies to optimize patient-centric care delivery effectively.

This paper contributes to advancing patient-centered care by applying a ST approach to identify strategic intervention opportunities within healthcare settings. It highlights the use of CLDs and SD modeling to address dynamic complexities, offering innovative solutions for improving patient engagement, satisfaction, and patient health outcomes.

## 2. Methodology

A SD and causal model were utilized to simulate the synthetic data for the analysis. Synthetic datasets, for which we propose the term synthsets, are not a novelty but have become a necessity. Although they have been used in computer vision since 1989, helping to solve the problem of collecting a sufficient amount of annotated data for supervised machine learning, the intensive development of methods and techniques for their generation belongs to the last decade [16]. This approach provides a controlled environment to analyze complex systems, as it eliminates the noise, outliers, and inconsistencies often present in raw datasets. By ensuring data quality and uniformity, synthetic data allow researchers to focus on system-level interactions and relationships without the distractions of irrelevant or incomplete information. While it provides significant advantages in terms of flexibility, consistency, and ethical considerations such as avoiding privacy concerns, this may not fully capture the unpredictability or diversity of real-world scenarios. Therefore, it is often used as a starting point for analysis, with future validations incorporating real-world data to enhance accuracy and applicability. We utilized the Vensim PLE 9.2.3 (Ventana Systems Inc., Harvard, MA, USA) [17] simulation platform to develop a SD model that integrates qualitative and quantitative data from various articles to establish the patient-centric paradigm. In the initial phase, we conducted in-depth research focused on collecting qualitative data. These data were instrumental in constructing a CLD that identifies the key factors influencing the improvement of patient-centric care. Subsequently, the qualitative data were combined with quantitative data to refine and develop the SD model for simulating patient behaviors and outcomes within the population.

## 3. Causal Loop Models

CLDs are used within ST and SD and have been utilized in population health since the 1970s. The focal areas of application have encompassed the following:

- Interactions between the capacity of healthcare settings or public health infrastructure and the epidemiology of diseases [18].
- The capacity and delivery of healthcare services in various domains, including population-based health maintenance organization planning, mental health, and the impact of natural disasters or acts of terrorism [19].
- The movement of patients within emergency and extended care settings [20].

**Figure 2:**
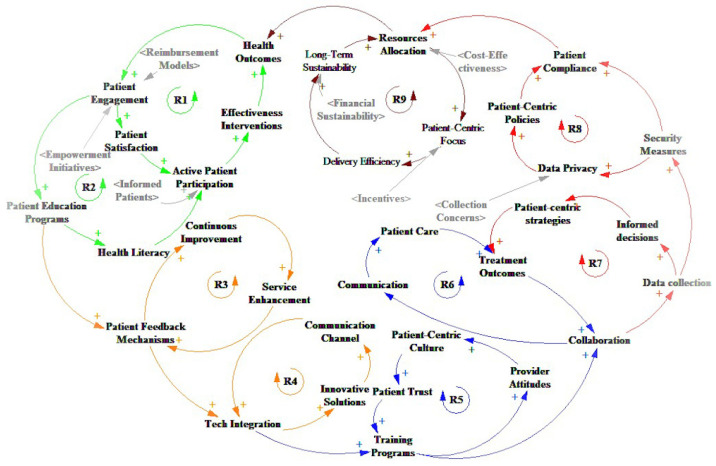
The causal loop model for patient-centric approaches in healthcare.

The complexity of patient centricity in the healthcare system arises from intricate interactions among various factors at different stages. A CLD depicted in Figure 2 is constructed to capture the multifaceted influences on patient centricity comprehensively. Grounded in ST, this model highlights dynamic interactions, causal relationships, and feedback loops, offering a comprehensive framework for understanding healthcare complexities. It serves as a paradigm, providing valuable insights into the factors influencing patients’ willingness and motivations within the healthcare system. The patient-centered perspective clarifies group dynamics and essential relationships, visually depicting causality and complexity in healthcare settings. All causal loop factors are derived from validated empirical studies that integrate both quantitative and qualitative research findings. Color-coded arrows represent specific causal loops, with reinforcing loops clearly labeled in corresponding colors for enhanced clarity and interpretation.

The factors linked predominantly by the green arrow examine patients’ intentions influenced by perceived usefulness and ease of use. Involving patients in their healthcare is crucial for enhancing clinical outcomes and elevating patient satisfaction. Research assessing patients’ inclination to participate in decision-making has yielded conflicting findings. The evidence supporting positive outcomes for patients actively involved in decisions about their medical care is indicative rather than definitive [21]. The U.S. government is actively promoting the adoption of Electronic Health Records (EHRs) through HealthIT.gov and offering incentives to physicians who utilize EHR meaningfully to reduce medical errors and enhance care quality. Contract Research Organizations (CROs) and independent service providers are developing educational websites accessible via mobile devices or the Internet to inform patients about various diseases [22].

Figure 2 shows the CLDs that shows how patient-centric care is improved through patient engagement, satisfaction, participation, practical education programs, outcome-informed practices, and empowerment initiatives. Patient-centric care is personalized treatment plans that lead to better patient health outcomes and informed decision-making, creating a responsive and empowering healthcare system.

According to Bosworth [23], comprehending the demand for healthcare services is pivotal for effective health services management. The U.S. Institute of Medicine defines quality as the “degree to which health services increase the likelihood of desired health outcomes and align with current professional knowledge [24]”. High-quality care, including safety, knowledge, effectiveness, efficiency, equality, and patient-centeredness, requires preserving safety, efficacy, patient-centeredness, timeliness, efficiency, and equity in healthcare [25]. Patient-centeredness, a key component, involves effective communication, elevating patient self-efficacy, enhancing trust, and supporting shared decision making [26]. This approach, promoted for improvement, is deemed an individualized and empowering care delivery method.

The proliferation of portable and wearable devices in the personal healthcare sector signals a positive trend towards self-empowerment for a healthier lifestyle. Orange arrows in the reinforcing loops (R3 and R4) represent this transformation, which includes the service creation, iterative correction, smooth tech integration, and continuous improvements shown in Figure 2. These innovations fortify patient-centric approaches, enabling efficient health condition tracking through telecare. The Internet of Things (IoT) is pivotal in remote vital sign monitoring and innovative solutions for chronic disease management, contributing to a holistic patient-centric healthcare paradigm.

Patients emerge as the driving force in digital health innovation in today’s digitally integrated world, leveraging their experiences to shape novel technologies (Figure 2, labeled in red arrows). Digital health aims to enhance patients’ experiences within the healthcare system and improve health outcomes [27]. Digital health technology tools (DHTTs) are crucial in remotely collecting health and disease-related data, often in clinical research studies. These tools encompass active tests, conducted through devices like tablets and smartwatches, and passive monitoring using smartphones, wearables, and beacons [28]. The rise of digital empowerment allows patients to take charge of their well-being through online tools, peer-to-peer sharing, health devices, and mobile apps [29]. Privacy, a paramount concern, is upheld in patient-centric environments, enabling informed decisions on health information sharing [30]. Health providers ensure privacy through risk analysis and compliance with legal frameworks like the Health Insurance Portability and Accountability Act (HIPAA) [31]. Collaborative efforts between health providers and consumers are vital for safeguarding patients’ protected information [29].

In addition, the healthcare system operates within a reinforcing loop as illustrated in Figure 2, which strives for optimization, with wine-colored arrows serving as indicators. This involves effective resource allocation, a patient-centric focus, streamlined delivery processes, long-term sustainability considerations, and incentivizing healthcare providers. Adopting this holistic approach ensures efficient resource utilization, provides transparent and high-quality patient care, maintains streamlined delivery processes, achieves long-term sustainability goals, and motivates healthcare providers through incentives. The interaction of these variables produces a dynamic equilibrium that improves healthcare performance overall and is consistent with the more general goals of sustainability, patient pleasure, and efficiency. In tandem, the U.S. government actively promotes the adoption of EHR through HealthIT.gov, offering incentives to doctors for meaningful use to reduce medical errors and enhance care quality. Within hospital management, a pivotal process involves health plans [22]. The “authorization” process, part of the balancing loop, reviews service requests and approves resources. It determines whether a patient’s health insurance covers treatment costs and offers patients the choice to self-pay in case of denial, emphasizing transparency and patient involvement in healthcare decision-making [32].

## 4. Model Implementation and Analysis

We utilize the analysis and implementation using system dynamics (in Vensim) and Python programming language. Potential system changes can be simulated first to predict their impact on performance. This research adopts a comprehensive approach, relying solely on a SD modeling design. This method enables the exploration and simulation of the intricate relationships within the healthcare system. By emphasizing SD modeling as the exclusive methodology, the study aims to provide a nuanced understanding of how interventions impact patient outcomes and system behavior. Data for the study are exclusively derived from simulated data, facilitating the creation of a robust foundation for SD modeling. The analytical framework centers on SD modeling techniques, eliminating the need for traditional statistical methods. Scenario analysis within the modeling process is employed to simulate and assess the consequences of various interventions on patient outcomes and the broader performance of the healthcare system. This singular emphasis on SD analytics ensures a focused exploration of the dynamic interplay within the healthcare system. Moreover, Python programming language was deployed for analysis and visualization.

### 4.1. Research Stocks, Flows, Variables, and Interventions Tabulation

This section uses tables to explain the stocks, flows, variables, and interventions of the SD model in the research. The SD building blocks are stock, flows, variables, parameters, and various auxiliaries. Stocks are accumulative amounts or states that take on a defined value with each transaction. In Table 1, data privacy and care coordination are independent stocks due to their vital roles in healthcare outcomes. Data privacy affects patient trust and willingness to share information, measured by compliance with regulations such as HIPAA and patient-reported trust. Care coordination reflects the integration of healthcare services, improving efficiency and patient experiences through timely care and effective communication. Both stocks capture their unique contributions to patient engagement and system performance. Other stocks, such as Active Patient Participation and patient engagement, represent the human aspect of healthcare. These measure patient involvement in care decisions and health management, influencing satisfaction and outcomes. Stocks like patient satisfaction, communication effectiveness, Quality of Care, resource allocation, and patient health outcomes represent the service quality and systemic components, ensuring a holistic evaluation of patient-centric care and its impact on healthcare performance.

The rates at which quantities change over time are referred to as flows. They are the model’s mathematical derivatives prescribing how the stocks, or cumulative amounts, increase or decrease. Flows are typically classified as inflow (which raises the stock over time) or outflow (which diminishes the stock over time). In Table 2, the model flows capture the dynamic interactions and feedback loops essential to a patient-centric healthcare settings. For instance, Improved Engagement and Enhanced Patient Engagement focus on increasing patient involvement in their care, leading to better adherence, satisfaction, and outcomes. Trust Development and enhanced patient–provider communication emphasize building strong relationships and clear communication, fostering trust and informed decision-making. Flows like Satisfaction Enhancement and Satisfaction Feedback ensure continuous improvements in care by incorporating patient experiences and feedback into service delivery. In addition, the impact of coordination on quality and insights for improved care highlight the importance of effective care coordination and data-driven insights to maintain high-quality, efficient, and adaptive healthcare services. These flows collectively reflect how interconnected processes drive improvements in patient outcomes and healthcare system performance.

Furthermore, the auxiliary variables in Table 3 represent critical factors influencing healthcare outcomes and equity. These include patient-centric factors such as patient trust, training programs, and collaboration, which are essential for engaging patients and improving care quality. Challenges such as Poor Communication with Healthcare Providers and Limited English Proficiency reflect gaps in patient–provider interactions, which can improve treatment adherence and outcomes. Environmental factors like Poor Air and Water Quality further illustrate external contributors to health disparities, showcasing the multifaceted nature of healthcare challenges.

Components in SDOH such as poverty, unemployment, and school segregation highlight systemic barriers to accessing healthcare. The SDOH are represented as a combined variable in the model, encapsulating multiple factors such as income, education, housing, and access to resources. By consolidating these elements under a single SDOH variable, the model reflects their collective impact on patient-centric approaches. This unified representation highlights the direct relationship between SDOH and patient-centric care, where changes in SDOH directly influence patient engagement, satisfaction, and overall health outcomes. Each component within the SDOH variable directly impacts the effectiveness of patient-centric interventions. For instance, lower income or limited education may hinder patient engagement, while stable housing and access to nutritious food can enhance adherence to care plans. By treating SDOH as a single interacting variable, the model captures its overarching influence on patient-centric dynamics, simplifying the analysis while maintaining its relevance. Future research can expand on this approach by exploring the sensitivity of patient-centric outcomes to variations in the SDOH variable, providing deeper insights into the interconnectedness of these factors. This approach ensures that SDOH is both integral to the model and aligned with its systemic impact on patient-centric healthcare strategies.

In the current model, we employed a subset of the interventions listed in Table 4, focusing on those most directly relevant to the primary objectives and scope of this study. Interventions such as Access to Healthcare Improvements, Community Health Education, and Patient-Centric Culture Interventions were prioritized for integration due to their immediate and measurable impact on patient engagement, satisfaction, and health outcomes. These interventions allowed us to validate the model’s structure and assess its capability to simulate real-world dynamics effectively. The choice of this subset reflects the study’s goal to create a foundational understanding of system dynamics in healthcare, with a focus on improving care delivery and patient experiences.

While interventions like Nutrition and Food Security Initiatives and Cultural Competency Training are not explicitly modeled in this iteration, they are recognized as critical factors influencing healthcare outcomes. These interventions address broader social determinants of health, such as food insecurity and cultural barriers, which significantly impact patient access, trust, and adherence. Although excluded from the current model due to scope limitations, these and other interventions hold significant potential for future iterations of the model. In subsequent studies, we plan to expand the framework to incorporate a more comprehensive range of interventions, enabling a deeper exploration of their individual and collective impacts on healthcare systems. This iterative approach allows for the gradual enhancement of the model’s robustness and adaptability, ensuring its continued relevance to diverse healthcare contexts and challenges. The tables in this section enable the comfort of identifying the parameters in the SD model.

### 4.2. The Stock and Flow Model for the Research

**Figure 3:**
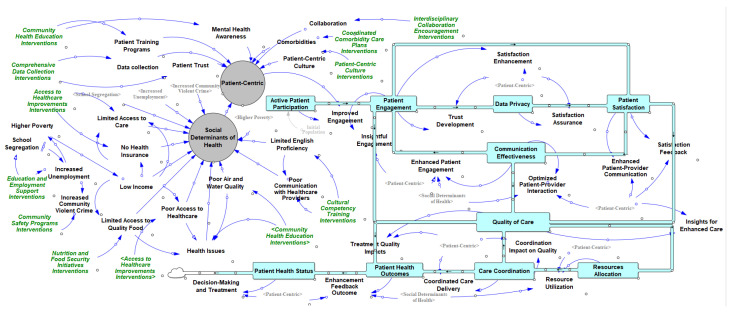
The system dynamics model using stocks and flows.

The research involves the modeling and simulation of the system, along with various variables. The stock and flow model is utilized to gain a deeper understanding of how interacting factors influence the overall dynamics. Table 5 presents Vensim’s setting parameters in the modeling process. The parameters in Table 5 were carefully chosen to ensure that the SD model accurately reflects the complexities of healthcare systems while maintaining computational efficiency. The initial time (2022), and final time (2032) establish a 10-year simulation period, allowing the model to capture both short-term impacts and long-term trends of healthcare interventions, such as improvements in patient engagement and satisfaction. The time step (0.0078125 years, or approximately three days) enables a high-resolution analysis of dynamic interactions and feedback mechanisms. This granularity is essential in healthcare systems, where changes often occur rapidly, such as the immediate effects of communication or education initiatives on patient outcomes. The use of “Year” as the unit of time ensures consistency in interpreting the results while still allowing detailed calculations within each year. The Euler integration method is applied to approximate system changes over each time step, offering a computationally efficient and stable approach to modeling continuous SDs. These parameters align closely with healthcare settings, where rapid changes, interdependence, and delayed effects are common. The fine granularity of the time step ensures that short-term variations are captured, while the longer simulation period provides insights into the sustained impact of interventions. To strengthen the methodological foundation, this study draws on previous research that successfully utilized SDs and CLDs in healthcare such as studies that used ST to address the unintended consequences of healthcare policies [8], research that emphasized the value of system dynamics in managing healthcare complexities and shaping policies [15], and studies that demonstrated the effectiveness of system dynamics in supporting clinical system transformations [6]. Additionally, Figure 3 shows the main SDs model of the research. This model involves the previous CLDs and the combination of stock and flow parameters to deeply comprehend the research’s complex and dynamic nature. This model considers all the variables involved in the impact of patient-centric care and the possible interventions to alleviate the adverse health outcomes of the interactions of the factors.

### 4.3. System Dynamics Model Equations Analysis

The equations describe the core principles of the SD modeling, specifically the interaction between stocks (S), flows (I for inflow and O for outflow), and time (t). These equations mathematically represent how stocks accumulate or deplete based on the net effect of inflows and outflows over time.

Differential Equation

The differential equation expresses the rate of change of a stock over time:

(1) dS/dt=I−O

Here, we have the following:

- *S* Represents the stock or state of the system at a given time (e.g patient engagement level, resource allocation).
- *I* The inflow rate, representing the rate at which the stock increases (e.g new patients engaged in healthcare programs per year).
- *O* The outflow rate, representing the rate at which the stock decreases (e.g., patients disengaging or leaving the healthcare system per year).
- The rate at which the stock changes over time is expressed as ds/dt, which is the inflow minus the outflow.

Integral Equation The integral form computes the stock value at a specific time by summing the net flow (inflow minus outflow) over a defined period:

(2) $$S\left(t\right)=S\left(t_{o}\right)+\int_{t_{o}}^{t}\left(I\left(T\right)-O\left(T\right)\right)dT$$

Here, we have the following:

- S(t) represents the stocks at a given time t.
- S(to) represents the initial value of the stock at the starting time to.
- I(T)−O(T) represent the inflow and outflow at time t, respectively, T.
- The integral $\int_{to}^{t}\left(I\left(T\right)-O\left(T\right)\right)dT$ represents the accumulated net flow (inflow minus outflow) over the period from S(t) to t.

In the integral form, we start with an initial stock level S(t) and add (integrate) all the net inflows from S(t) to t. This gives us the stock level at time t. If the inflows exceed the outflows during this period, the stock will increase; if the outflows exceed the inflows, the stock will decrease.

### 4.4. Design Motivation and Application

The equations were designed to model dynamic systems where variables evolve over time due to feedback and interdependencies. In this study, the goal is to track and analyze patient healthcare outcomes, such as patient engagement, communication effectiveness, or resource utilization, by quantifying how interventions or systemic changes impact these stocks.

- Predicting the long-term effects of interventions on patient engagement;
- Assessing how resource allocation impacts patient healthcare outcomes;
- Evaluating the sustainability of healthcare initiatives over time.

Example: Patient Engagement Dynamics Consider a scenario where the stock S(t) represents the level of patient engagement:

- Initial engagement S(to): 1000 patients engagement in 2022.
- Inflow I(t): 100 new patient engage per year.
- Outflow O(t): 50 patients disengage per year.

Using the differential equation

(3) dS/dt=100−50=50patients/year

Using the integral equation for a period from 2022 to 2025 (to = 2022, t = 2025),

(4) $$S\left(2025\right)=S\left(2022\right)+\int_{2022}^{2025}\left(I\left(100\right)-O\left(50\right)\right)dT$$

(5) S(2025)=1000+3×50=1150patients

This example demonstrates how the equations project the system’s behavior over time, providing actionable insights for decision-making in healthcare strategies.

## 5. The Application of Python System Dynamics in the Research

**Figure 4:**
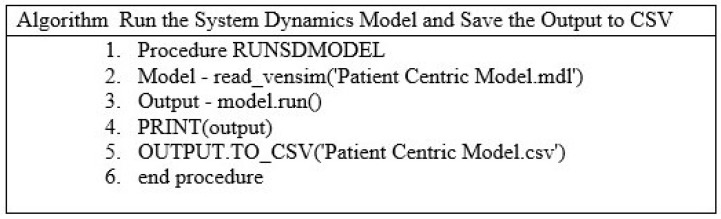
The procedure of loading the system model.

This section provides a detailed explanation of the Python System Dynamics (PySD) used in the study. The following is the step-by-step breakdown of the pseudocode as shown in Figure 4:

- Procedure RUNSDMODEL: This is the declaration of the procedure or function. It encapsulates the steps required to run the system dynamics model and export its results for further analysis.
- Model = read-vensi(‘Patient Centric Model.mdl’): This line initializes the SDs model by loading the specified Vensim model file (Patient Centric Model.mdl). The read-vensim() function is used to interface with the Vensim software, allowing the model to be translated into a format that can be executed programmatically (e.g, in Python).
- Output = model.run(): This step executes the loaded model. The run() method simulates the dynamics of the system based on the specified parameters, equations, and initial conditions defined in the model file. The output typically contains time-series data of the system’s stocks, flows, and other variables over the simulation period.
- PRIT (output): This line outputs the simulations of the results to the console or terminal, providing an immediate view of the model’s outcome.
- OUTPUT.TO_CV(‘Patient Centric Model.csv’): This step exports the simulation results (output) to a CSV file for further analysis and visualization and the file name Patient Centric Model.csv specifies where the data will be saved.
- end procedure: This marks the conclusion of the pseudocode.

The Vensim-based SDs model can be translated and launched in a Python environment more easily with the PySD module. The dynamic model manipulation is made easier to use by the library’s ability to read Vensim models and translate them into Python scripts. It also assured compatibility with several Python libraries. Additionally, Pandas is another Python gem that improves data handling by allowing users to creatively manipulate information through its flexible DataFrame and Series structures. Combining numerical accuracy with flexible data management, NumPy and Pandas work together to form a powerful team for thorough data analysis [33]. The scripts and introduction of the Matplotlib library were written by [34]. This library is used to create graphs. Typically, the Seaborn library is utilized to improve data visualization. Compared to Matplotlib, it is a more visually appealing, informative, and intuitive tool.

Using the algorithm as shown in Figure 4, the Patient Centric Model.mdl, a simulation file, is launched. It applies the read-vensim SD model from the Vensim software. The output variable, which is saved with the model’s running result by the model.run function, shows the dynamics of the model over time. This result allows the programming process’s model to be rapidly assessed. Furthermore, the output is kept in a csv file with the name Patient Centric Model.csv.

## 6. Results and Discussions

This section explores how the graphical and tabular results from the SD modeling and analysis should be interpreted. Comprehensive discussions about the impact of these outcomes as they are derived from the models are also included. The data are exclusively derived from simulation, as conducting experiments with real-world data would require a significant amount of time to collect. For now, we rely on synthetic data to carry out the analysis. The outputs associated with the trend identified during the exploratory analysis are covered in detail in the following subsections.

### 6.1. The Model Analysis

The projections stem from the simulated impact of patient-centric approaches, which emphasize improved engagement, satisfaction, and communication effectiveness as key drivers of better patient health outcomes. The trends also reflect the interplay between targeted interventions and social determinants of health, revealing how accessibility, resource allocation, and systemic disparities shape the dynamic evolution of healthcare performance over time.

#### 6.1.1. The Patient Engagement Analysis

**Figure 5:**
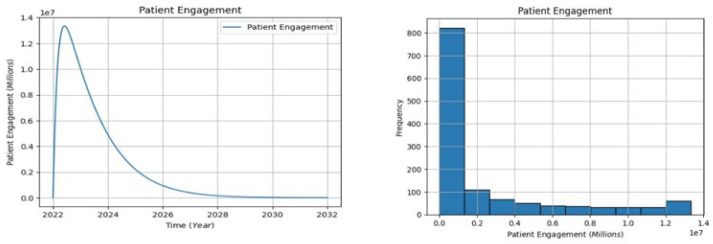
The patient engagement visualization.

The graph in Figure 5 shows a visual representation of the evolving trend of patient engagement in population in the United States healthcare system over time, with key observations including an initial increase from 0 in 2022 to approximately 1.4 million between 2022 and 2023. However, this upward trajectory subsequently declines to around 0.0 million by the year 2032. Examining the distribution, the first bar indicates that the majority of the patient engagement in the population falls within the 0–0.1 million range, while the second bar represents the lowest standard of the patient population, ranging from 0.2 to 1.3 million. The left-skewed distribution of the graph suggests that a larger proportion of patients exhibit higher engagement levels, influenced by patient-centric approaches and social determinants of health (SDOH) in the United States. Despite the overall positive trend, the analysis underscores existing disparities, emphasizing that not all patients participate equally or have uniform access to healthcare resources. To address these disparities, it is recommended that healthcare systems focus on targeted efforts such as improving access, promoting patient-centric care, and addressing SDOH. These interventions can contribute to enhancing patient engagement and achieving more equitable patient health outcomes across diverse patient populations.

#### 6.1.2. The Patient Satisfaction Analysis

**Figure 6:**
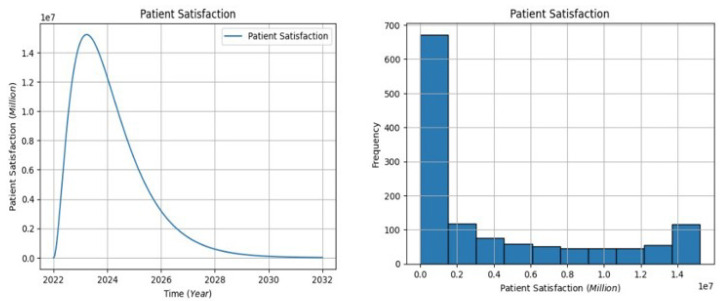
The patient satisfaction visualization.

The line plot in Figure 6 shows the U.S. patient satisfaction populations’ progression from 2022 to 2032. The annual peak for new cases occurs between 2023 and 2032, with over 1.55 million cases reported. This positive development is attributed to the expansion of healthcare access, which has been facilitated by patient-centered care and the SDOH. Satisfaction does, however, continue to decline from 2024 to 2032. Given that the distribution is concentrated in the 0.0 million to 0.19 million range, a sizable portion of patients likely have annual satisfaction levels that are noteworthy. Conversely, the bottom bar, which ranges from 0.2 million to 1.4 million, represents fewer common instances of satisfaction. Because of the left-skewed distribution, a larger percentage of people report higher levels of satisfaction. In conclusion, the report highlights the favorable trends in patient happiness and healthcare access in the United States, recommending that access enhancement, patient-centric care, and SDOH be addressed going forward to achieve long-term gains.

#### 6.1.3. The Communication Effectiveness Analysis

**Figure 7:**
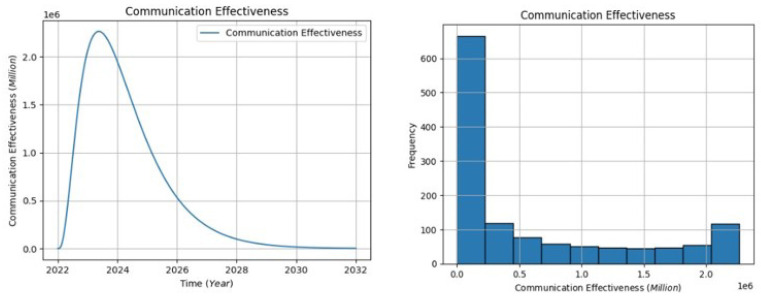
The communication effectiveness visualization.

Figure 7 The projected communication effectiveness shows an initial increase, reaching a peak of approximately 2.4 million patient populations between 2022 and 2024, starting from a baseline of 0. However, a gradual decline ensues from around 2024 to 2032, indicating a diminishing effectiveness of communication between patients and healthcare providers. The distribution of communication effectiveness is portrayed on a graph with the horizontal axis representing the population impacted by communication, and the vertical axis denoting the frequency of these populations. The highest frequency falls between 0.0 and 0.23 million, while the lowest is between 0.23 and 2.5 million. The left-skewed distribution implies that higher populations experience more effective communication with healthcare providers. The declining trend could be attributed to various factors such as evolving technologies, shifting healthcare dynamics, or changing patient expectations. To address this decline, healthcare systems should consider continuous training for effective communication, the integration of advanced communication technologies, and regular assessments of patient–provider interactions. These interventions can contribute to sustaining and enhancing communication effectiveness over time.

#### 6.1.4. The Patient Health Outcomes Analysis

**Figure 8:**
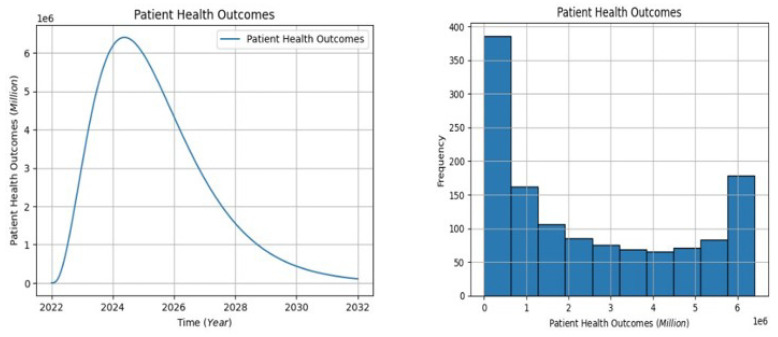
The patient health outcomes visualization.

In Figure 8, the projection indicates an initial increase, peaking at approximately 6.8 million patient populations between 2022 and 2025, starting from a baseline of 0. However, a subsequent gradual decline occurs from around 2025 to 2032, signaling a reduction in overall patient-centric outcomes. The distribution of patient outcomes on the graph portrays the population on the horizontal axis and the frequency on the vertical axis. The highest frequency falls between 0.0 and 0.8 million, with the second-highest bar at around 6 million and the third-highest at 1 million. A gradual drop occurs from 1.2 to 5.8 million. The left-skewed distribution suggests that higher populations experience satisfaction with patient health outcomes. To enhance patient health outcomes, incorporating patient-centric approaches and addressing SDOH is crucial. Interventions should focus on personalized care, health education, and community-based initiatives. Healthcare systems can implement regular assessments of patient needs, promote preventive care, and collaborate with social services to address underlying determinants affecting health. This holistic approach ensures that interventions are tailored to individual patient needs, fostering improved and equitable health outcomes across diverse populations.

### 6.2. Implementing Interventions’ Impact on Patient-Centric and SDOH in Populations over Time (Sensitivity Analysis)

This section offers a detailed examination of the model’s sensitivity analysis, which involves methodically varying different input intervention parameters. The objective of this procedure is to clarify the effects of various treatments in the complex interaction between patient-centered methods and SDOH. The next subsections go into great detail into the particular effects that these interventions have on the dynamics of the model.

#### 6.2.1. Access to Healthcare Improvements Impact on Patient Engagement

Interventions about the availability of health metrics are tactics or initiatives meant to enhance, evaluate, and promote the well-being of individuals or groups of people. In line with the view of the United Nations, health is acknowledged as an essential human right. Improving access to healthcare plays a major role in improving overall health and mitigating disease. Accessibility issues in low-income nations are primarily related to the availability of basic health services, such as having a doctor consultation or receiving proper care during pregnancy and childbirth. This right emphasizes several rights related to the prerequisites for advancing and protecting health. These include protecting people from abuse and cruel treatment, encouraging lifestyles and living situations that reduce the risk of illness, accidents, and injuries, and guaranteeing access to both preventive and curative health services [35].

Patient engagement, involving the active participation of patients and their families in healthcare, is endorsed by mandates from organizations like the Joint Commission [36] and the World Health Organization [37]. Despite challenges in patient willingness and power dynamics, the majority desire involvement. Recognizing patients as vital partners, the Department of Health and Human Services emphasizes patient engagement in its strategy for preventing adverse drug events, aligning with principles of equity and contributing to the creation of safer healthcare systems [38].

**Figure 9:**
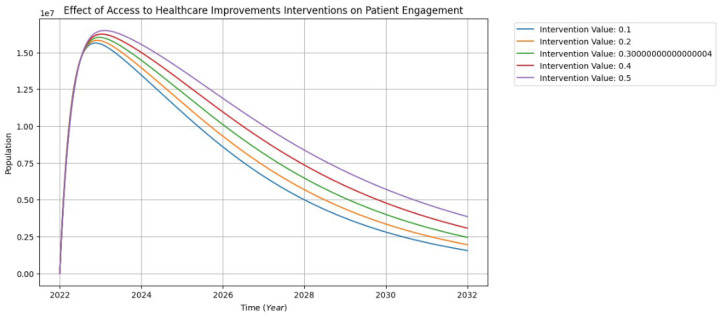
The interventions of patient engagement visualization.

The effort required to address the effects of patient-centric and SDOH factors on patient engagement populations is quantified by the values of the interventions shown in the graphs in Figure 9 below. The various intervention numbers (0.1, 0.2, 0.3, 0.4, and 0.5) represent the proportionate impact on these groups. For instance, 0.1 is equivalent to 10%, 0.2 to 20%, 0.3 to 30%, 0.4 to 40%, and 0.5 to 50% of the intervention value as shown in Figure 9.

Figure 9 illustrates the impact of interventions aiming to enhance access to healthcare on patient engagement populations. The interventions initially exhibit latent influences from 2022, ranging from 0.0 to approximately 1.50 million in the population. A discernible peak of around 1.6 million occurs between 2022 and 2023, reflecting heightened effects in line with increased patient engagement. However, a subsequent decline from 2024 to 2032 may be attributed to factors such as poor communication with healthcare providers, a lack of a patient-centric culture within the healthcare system, and the presence of comorbidities among patients. Recognizing and addressing these influences is vital for sustaining positive intervention impacts and ensuring enduring patient engagement in healthcare practices.

#### 6.2.2. Access to Healthcare Improvements Impacts on Patient Satisfactions

Patient satisfaction is becoming a crucial component of healthcare providers’ financial performance as well as their patients’ health as a result of the shift in healthcare systems from fee-for-service to alternative payment models and the growth of consumer healthcare options [39].

**Figure 10:**
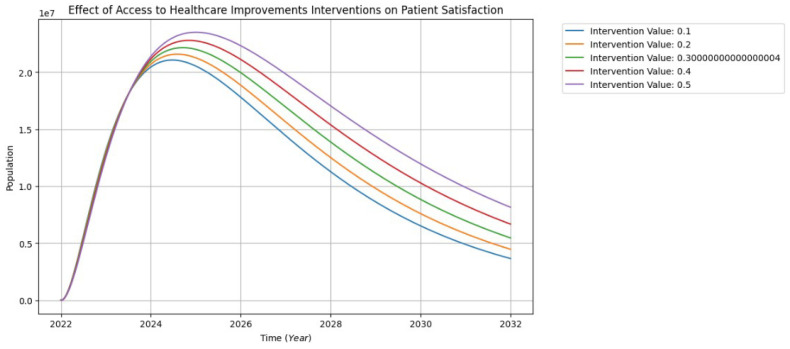
The interventions of patient satisfaction visualization.

Figure 10 illustrates the impact of access to healthcare improvement interventions on various aspects of patient satisfaction, encompassing active participation, engagement, trust, data privacy, data collection, and patient training programs, over 10 years. Beginning in 2022, SDOH and patient-centric approaches catalyze a rapid rise in patient satisfaction from 0 to 2.4 million between 2022 and 2025. However, a phase of limited significant effects follows, characterized by narrower curves. Subsequently, the model projects a substantial increase in the population, with the 50% intervention demonstrating the most significant rise, and the 10% intervention displaying the least impact. From 2026 to 2032, a gradual decline in the patient satisfaction population to approximately 0.4 million is projected. Interestingly, despite the decline, there are concurrent rises in the influences of interventions. The notable divergence between the 50% and 10% interventions suggests that additional targeted efforts, especially at higher levels, may be instrumental in alleviating the increase in population and sustaining positive trends.

#### 6.2.3. Access to Healthcare Improvements Impacts on Communication Effectiveness

Effective communication is paramount in healthcare teams, fostering collaboration among diverse professionals and ensuring patient-centered care. In these teams, information exchange is crucial for coordinated efforts, requiring cultural and linguistic competence to address diverse backgrounds. Sociolinguistic practices, such as clear and context-appropriate communication, break down hierarchical barriers for open dialogue. Patient involvement is emphasized, necessitating transparent communication about conditions and treatment options. The quality of communication directly impacts patient outcomes, reducing errors and enhancing satisfaction. Overall, dynamic and deliberative communication within healthcare teams is essential for achieving shared goals, delivering optimal patient care, and improving overall health outcomes [40].

**Figure 11:**
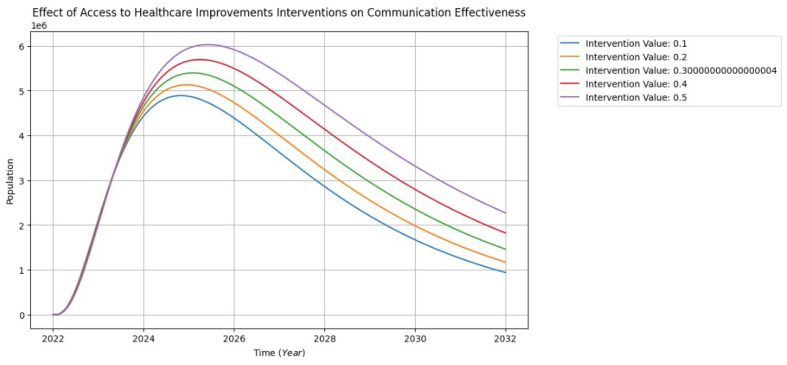
The interventions of communication effectiveness visualization.

Figure 11 depicts the impact of access to healthcare improvement interventions on various aspects of communication, emphasizing collaboration among healthcare professionals, over 10 years. Commencing in 2022, SDOH and patient-centric approaches have driven a rapid surge in communication, escalating from 0 to 6 million between 2022 and 2026. However, from 2027 to 2032, the model projects a gradual decline in communication across the population to approximately 1 million. Intriguingly, despite the decline, there are simultaneous rises in the influences of interventions. The noteworthy divergence between the 50% and 10% interventions suggests that additional targeted efforts, particularly at higher levels, may prove crucial in mitigating the decline and sustaining positive trends in communication among healthcare professionals.

#### 6.2.4. Access to Healthcare Improvements Impacts on Patient Health Outcomes

Improving healthcare outcomes necessitates an increased investment of time by physicians in patient interactions. The participation of the teaching physician should be marked by enthusiasm, motivation, and responsiveness to individual patient needs. A robust and hearty engagement between patients and physicians is crucial for society to reap the benefits of physician health education. This involves fostering a deep understanding among patients about the profound impact that healthy interventions can have on both present and long-term health. Building meaningful connections and facilitating comprehensive communication between physicians and patients is essential for empowering individuals to actively participate in their healthcare, promoting informed decision-making, and ultimately enhancing overall health outcomes [41].

**Figure 12:**
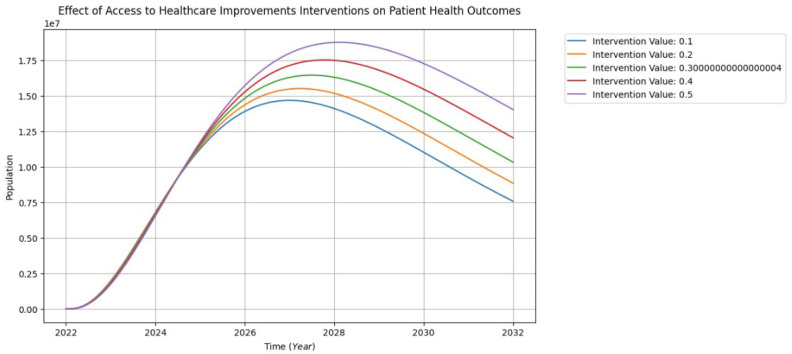
The interventions of patient health outcome visualization.

In Figure 12, commencing from 2022 on the graph, the intervention aimed at improving access to healthcare exhibits an initial 10% influence, resulting in a decrease in the patient health outcomes population from approximately 1.25 million in 2025 to about 0.75 million by 2032. During this period, the intervention effects appear latent. Subsequently, with incremental increases in interventions (20%, 30%, 40%, 50%) from 2028, there is a visible uptick in the population, peaking and gradually declining from 2030 to 2032.

These intervention processes signify an increase in patient health outcome populations due to the influence of various measures. Suggestions for further improvement include enhancing patient satisfaction, fostering effective communication with healthcare providers, optimizing resource allocation, ensuring the quality of healthcare services, promoting seamless care coordination, upholding data privacy standards, and actively engaging patients in their healthcare decisions. These elements collectively contribute to an environment conducive to improved patient outcomes, emphasizing the multifaceted nature of healthcare interventions.

The declining trends in engagement and outcomes observed in the graphs can be explained by the dynamic interactions and feedback loops within the modeled healthcare system. While initial interventions often lead to improvements in key factors such as patient engagement, satisfaction, and health outcomes, these gains may diminish over time due to systemic constraints and unintended consequences. For instance, as patient engagement increases, the demand for healthcare resources and provider attention may exceed system capacity, leading to bottlenecks and inefficiencies. Similarly, social determinants of health, such as limited access to care or economic disparities, may counteract the positive effects of interventions, further exacerbating declines. The interconnected nature of variables such as trust, communication effectiveness, and resource allocation creates complex feedback loops that can amplify negative trends if not properly managed. These declines highlight the importance of addressing systemic issues and ensuring that interventions are designed to adapt dynamically to the demands of the evolving system. Future iterations of the model can explore additional strategies and interventions to mitigate these challenges and sustain positive outcomes over the long term.

## 7. Discussion

A key limitation of this study is the reliance on simulated data, which, while providing a controlled environment for exploring dynamic interactions, cannot fully replicate the complexities of real-world healthcare settings. Simulated data simplify certain aspects of healthcare dynamics and may not capture critical nuances, such as resistance to interventions by patients, providers, or organizations. These factors, including patient hesitation, provider workload burdens, and organizational constraints, are crucial to determining the success of interventions and can lead to outcomes that differ in practice.

Additionally, the model does not account for external economic, regulatory, or policy factors that significantly influence healthcare systems. Changes in funding, policy reforms, economic shifts, or broader social determinants could impact the effectiveness of patient-centric interventions. The assumptions made in the simulated environment, while necessary, may oversimplify healthcare complexities. Future iterations should incorporate real-world data to validate findings, refine assumptions, and address external factors, enhancing the model’s relevance and utility for decision-making and policy development.

## 8. Conclusions

In conclusion, this research leverages a holistic system thinking approach, integrating patient-centric models, CLD, and SD modeling to address the inherent complexities of healthcare systems. By exploring patient engagement, satisfaction, communication effectiveness, and patient health outcomes over a 10-year period, the study provides deep insights and actionable recommendations to improve healthcare care delivery. The use of sensitivity analysis further enhances the findings, shedding light on the varying impacts of interventions and offering valuable guidance for policy influence and decision-making. In addition, the integration of innovative digital health tools underscores the evolving role of technology in promoting patient-centric care and improving patient health outcomes.

However, the study’s reliance on simulated data introduces a limitation in fully capturing the dynamics of healthcare in the real world, and the findings may not be entirely generalizable in diverse healthcare settings. Although the research offers a comprehensive 10-year projection, it does not extensively address long-term trends or external influences, such as economic or cultural factors. Practical challenges in translating theoretical insights into scalable, real-world interventions also remain. Despite these limitations, the study contributes significantly to understanding the dynamic interaction between patient-centric approaches, social determinants of health, and patient health outcomes. It emphasizes the critical need for sustained efforts to address disparities, improve access, and foster equitable, effective healthcare systems that adapt to the evolving needs of diverse patient populations.

## Figures and Tables

**Table 1:** The stocks employed in the model.

Model Stocks
Active Patient Participation
Patient Engagement
Data Privacy
Patient Satisfaction
Communication Effectiveness
Quality of Care
Resources Allocation
Care Coordination
Patient Health Outcomes
Patient Health Status

**Table 2:** The flows employed in the model.

Model Flows
Improved Engagement
Satisfaction Enhancement
Trust Development
Satisfaction Assurance
Satisfaction Feedback
Enhanced Patient–Provider Communication
Enhanced Patient Engagement
Insightful Engagement
Optimized Patient–Provider Interaction
Insights for Enhanced Care
Coordination Impact on Quality
Treatment Quality Impacts
Resource Utilization
Coordinated Care Delivery
Enhancement Feedback Outcome
Decision-Making and Treatment

**Table 3:** The auxiliary variables employed in the model.

Auxiliaries Factors
Patient-Centric
Patient Trust
Patient Training Programs
Mental Health Awareness
Collaboration
Comorbidities
Patient-Centric Culture
Data Collection
Social Determinants of Health
Limited Access to Care
Higher Poverty
No Health Insurance
School Segregation
Increased Unemployment
Low Income
Increased Community Violent Crime
Limited Access to Quality Food
Poor Access to Healthcare
Health Issues
Poor Air and Water Quality
Poor Communication with Healthcare Providers
Limited English Proficiency

**Table 4:** The interventions employed in the model.

Interventions
Access to Healthcare Improvements Interventions
Community Health Education Interventions
Comprehensive Data Collection Interventions
Interdisciplinary Collaboration Encouragement Interventions
Coordinated Comorbidity Care Plans Interventions
Patient-Centric Culture Interventions
Education and Employment Support Interventions
Community Safety Programs Interventions
Nutrition and Food Security Initiatives Interventions
Cultural Competency Training Interventions

**Table 5:** Model within time limits.

Parameter	Value
Initial Time	2022
Final Time	2032
Time Step	0.0078125
Units of Time	Year
Integration	Euler

## Data Availability

Data are contained within the article.
